# Racial and ethnic disparities in subjective cognitive decline: a closer look, United States, 2015–2018

**DOI:** 10.1186/s12889-021-11068-1

**Published:** 2021-06-24

**Authors:** Sangeeta Gupta

**Affiliations:** Department of Public and Allied Health Sciences, Delaware State University, 1200 N DuPont Highway, Dover, Delaware, 19901 USA

**Keywords:** Subjective cognitive decline, Behavioral risk factor surveillance system, Chronic conditions, Functional limitations, Blacks, Whites, Hispanics

## Abstract

**Background:**

Subjective cognitive decline (SCD), characterized by self-experience of deterioration in cognitive performance may be a precursor to Alzheimer’s disease (AD). Given the association of AD with dependence and disability for a long duration, earlier the detection, the sooner people and their families can receive information regarding better management. It is critical to explore disparities amongst racial and ethnic populations with SCD in order to facilitate targeted interventions. The primary objective was to identify disparities in prevalence of SCD amongst Whites, Blacks and Hispanics by select sociodemographic characteristics and functional limitations in a U.S. population-based sample of non-institutionalized adults aged 45 and older. The secondary objective was to assess the association between SCD and select chronic conditions (angina, heart attack, stroke, diabetes, high blood pressure and high cholesterol) by race/ethnicity.

**Methods:**

Combined data (2015–2018) were obtained from the Behavioral Risk Factor Surveillance System (BRFSS) to conduct a population -based study. Analyses included 179,852 respondents aged 45 years or older who answered the SCD screening question as “yes” (*n* = 19,276) or “no” (*n* = 160,576). Descriptive statistics examined sociodemographic characteristics including functional limitations amongst racial/ethnic groups with SCD. Association of SCD with chronic conditions by race/ethnicity was also calculated.

**Results:**

Overall, 10.8% (CI: 10.6–11.1) of adults aged 45 years or older reported SCD.10.7% Whites, 12.3% Blacks and 9.9% Hispanics experienced SCD. Blacks and Hispanics with SCD were more likely to be in the younger age group (45–54 years), less educated, low income, without access to health care, living alone and with functional limitations. Only half had discussed cognitive decline with a health care professional. Prevalence of selected chronic conditions was significantly higher in all racial/ethnic groups with SCD.

**Conclusions:**

Demographic trends predict a larger proportion of Hispanics and Blacks with SCD in the coming years. This information can lead to identification of opportunities for addressing negative SCD outcomes in minorities affected by inequitable conditions.

## Background

The United States population has become increasingly diverse. The proportion of racial and ethnic minorities has been growing steadily over the last decade. Minorities, classified as those of any race other than non-Hispanic, single-race Whites by the US Census Bureau, currently constitute about one third of the U.S. population and are composed of several different race categories—Black or African American, American Indian or Alaska Native Asian, Native Hawaiian or Other Pacific Islander. Hispanics are also considered a minority, although strictly speaking Hispanic, or Latino, is defined as an ethnicity rather than a race by the Census Bureau. July 2019 census figures show African Americans to be the largest racial minority, comprising an estimated 13.4% of the population and Hispanics as the largest ethnic minority, comprising an estimated 18.3% of the population [1].

According to the Pew Research Center, minority populations are projected to rise to 56% of the total U.S. population in 2060 [2]. By 2060, minority populations aged ≥65 years will represent 45% of the U.S. population, which is up from 22% in 2014. By 2060, the percentage increase in total population by race and ethnicity is estimated to be 75% for non-Hispanic Whites, 172% for African Americans, and 391% for Hispanics [1, 2]. As the population ages and becomes more diverse, the burden of age-related chronic conditions, such as Alzheimer’s disease and related dementias (ADRD) are projected to disproportionately impact minorities, both in terms of prevalence and severity [3–5]. Studies indicate that older Blacks and Hispanics are up to twice as likely to have ADRD as older Whites [5]. Minority patients with Alzheimer’s disease (AD) oftentimes present with greater cognitive impairment than their white counterparts. Not only are Hispanic and Black older adults at a higher risk for ADRD than non-Hispanic White older adults; research evidence shows there are disparities in the number of years in late life spent with dementia [4]. It is documented that Hispanics and Blacks at age 50 are estimated to spend a greater number of years with dementia and, consequently, a greater proportion of their remaining lifespan with lower quality of life. The number of years expected to be spent with dementia is approximately three times more for Blacks and 3.5 times more for Hispanics relative to Whites at age 50 [4, 5].

Burden of ADRD, a condition with escalating costs and very limited treatment options, is a growing public health problem for which early detection is critical. In 1982, Reisberg and colleagues [6] classified the course of Alzheimer’s disease progression and introduced the concept of Subjective Cognitive Decline (SCD), possibly one of the earliest detectable symptoms for dementia. SCD characterized by self-experience of deterioration in cognitive performance not detected objectively through formal neuropsychological testing, is considered among the earliest clinical manifestations of AD when individuals have sustained only mild neuronal damage and are able to functionally compensate [7]. SCD has garnered significant interest since it was first put forward. Some research findings indicate as many as 60% of individuals with SCD decline to AD over a 15-year period [8, 9]. Regardless of whether or not SCD leads to more significant cognitive disruption (ADRD, or another dementia), there is a great chance it will impact a person’s ability for self-care, activities of daily living and managing comorbid chronic conditions [10].

The construct of SCD came with a caveat. Since its inception, there has been much confusion in the extant literature about how SCD is formally diagnosed particularly its differentiation from subjective memory concerns [11]. The notion of SCD as a predictor for objective memory impairment and/or progression to ADRD faces an additional challenge in minority groups. Disparate burden of chronic stress and mental health conditions such as depression and anxiety in vulnerable communities may influence the experience of cognitive impairment. Reporting of subjective decline may also be influenced by cultural practices and language barriers [12]. In a community study of African Americans in Baltimore, SCD was found to be associated primarily with factors of psychological well-being, such as higher levels of perceived stress, unrelated to objective cognitive impairment [12]. In another community study in North Carolina that directly compared African Americans and Caucasians, African Americans were less likely to complain of deterioration in memory, despite greater evidence of objective cognitive decline [13]. In a clinic -based sample of Hispanic older adults, SCD was found to be associated with symptoms of depression rather than with concurrent cognitive performance [14]. Measures of cognition, especially subjective measures, appear to be racially biased in terms of efficacy.

Another reason to focus attention on minority populations relates to the severe limitations placed by their social and physical environment [15]. Health status including a person’s risk for dementia is strongly influenced by the environment in which people live, known collectively as the social determinants of health (SDOH). Minority communities are more likely to experience limited access to health care, lower level of education, less income and loneliness/ lack of social support. Research findings have shown a relationship between these factors and dementia [15, 16]. Differences in SDOH contribute to the stark and persistent disparities in other chronic diseases among racial and ethnic minorities. Disparate burden of certain disorders such as heart disease and diabetes that are associated with a higher risk for ADRD [16, 17], make these communities more vulnerable. Furthermore, SCD with a concomitant chronic condition(s) is likely to compound functional limitations and amplify barriers to treatment in these vulnerable communities.

Few population-based studies have explored SCD across race and ethnicity in detail [3]. In recent years, a few national studies have reported on SCD by race/ethnicity [17–20]. However, their findings were mostly limited to overall SCD prevalence. Disparities by sociodemographic characteristics, functional limitations and chronic conditions amongst racial/ethnic groups with SCD have not been explored in detail at the population level.

This study takes a closer look at ethnoracial disparities in cognitive decline using the Centers for Disease Control and Prevention (CDC) Behavioral Risk Factor Surveillance System (BRFSS) national data. The main objective of this study was to examine the prevalence of SCD by select sociodemographic characteristics and describe SCD associated functional limitations amongst non-Hispanic Whites and the two largest minority populations - non-Hispanic Blacks and Hispanics. Additionally, association of comorbid chronic conditions (angina, heart attack, stroke, high cholesterol, high blood pressure and diabetes) and social determinants (education, income, living alone and health care access) were also described among racial/ethnic groups with and without SCD.

Given persistent disparities in access to quality health care, not all population subgroups reap the benefits of public and private health ADRD initiatives, raising a critical public health issue, especially in lieu of the aging minority population [3–5]. Our hypothesis is that SCD subgroup is more strongly associated with cognitive risk factors such as chronic disease(s) and SDOH with a disproportionately higher burden in minority populations. Study results will support informed decisions and facilitate prioritization of resources for minority groups with SCD.

## Materials and methods

### Data source

Behavioral Risk factor Surveillance System (BRFSS) is the nation’s premier system of health-related random-digit-dialed, telephone surveys (landline and cell phone) of noninstitutionalized U.S. adults aged 18 years or older [21, 22]. The survey assesses data about health-related risk behaviors, chronic health conditions, and use of preventive services in all 50 states, District of Columbia (DC) and US territories [21]. A six-question module on cognitive decline asked of people aged 45 years or older included questions about SCD and associated difficulties was added as an optional module in 2015 [22]. BRFSS data for 2015 through 2018 (*n* = 1,756,645) were compiled into a common data set. A subset was created for participants aged 45 years or older (*n* = 1,274,754). Of participants aged 45 years and older,181,464 participants responded to the BRFSS Cognitive Decline module. Excluding 1612 participants with inadequate responses, data from 179,852 respondents aged ≥45 years were included in the analysis including 19,276 with SCD and 160,576 without SCD. The excluded respondents were similar to those included in the study in terms of age group and race/ethnicity but were more likely to have a college education.

All 50 states plus DC and Puerto Rico administered the SCD module at least once for years 2015 through 2018. Six states (Georgia, Hawaii, Mississippi. New Jersey, Oregon and Puerto Rico) administered the SCD module more than once in this time period. Only the most recent year’s data for these six states was included in these analyses.

Overall, BRFSS response rate varied from 47.1% in 2015 (ranged from 33.9 to 61.1); 47.0% in 2016 (ranged from 30.7 to 65.0%); 45.1%in 2017 (ranged from 30.6 to 64.1) to 49.8% (ranged from 38.8 to 67.2) in 2018 [22, 23]. Response rates for BRFSS are calculated using standards set by the American Association for Public Opinion Research (AAPOR) Response Rate Formula #4 [23]. The response rate is the number of respondents who completed the survey as a proportion of all eligible and likely-eligible people. For detailed information please see the BRFSS Summary Data Quality Report [23].

### Definitions

Racial categories for this analysis (non-Hispanic white; non-Hispanic Black; and Hispanic) are obtained from BRFSS data codebook [21, 22]. Ethnicity is determined by a ‘yes’ response to the question, “Are you Hispanic, Latino/a, or Spanish origin?” Racial categories presented in BRFSS are the same as the six US census categories (White, Black or African American, American Indians and Alaska Native, Asian American, and Native Hawaiian and Other Pacific Islander) as well as people of two or more races. BRFSS provides several race variables, allowing researchers to choose single race categories with a residual multiple race category or a recode that allocates multiple race individuals to a race category based on self-identified preferred race [24]. BRFSS variables for race used in this study are as follows: i) White - Non-Hispanic; ii) Black-Non-Hispanic and iii) Hispanic. Individuals reporting more than one racial category were excluded from this analysis.

A “yes “response to the BRFSS question, “During the past 12 months, have you experienced confusion or memory loss that is happening more often or is getting worse?” defined presence of SCD. BRFSS questionnaire further assesses SCD related limitations by asking the following: (i) how often SCD caused them to give up day-to-day activities such as cooking, cleaning, taking medications, driving, or paying bills; (ii) how often they needed assistance with these day-to-day activities; (iii) how often were they able to get the help they needed; and (iv) how often did SCD interfere with their ability to work, volunteer, or engage in social activities about the home; A response of “always, usually, or sometimes” to the above denoted the presence of SCD associated functional impairment. Further, proportion of adults with SCD who discuss their confusion or memory loss with a health-care professional were also calculated by a ‘yes’ response to the question, “Have they discussed their confusion or memory loss with a health care professional?”

Each selected race/ ethnicity category was further sub classified by age group (45–54;55–64 and ≥ 65 years), sex (male and female), highest level of education (did not graduate high school; graduated high school or equivalent; attended college or technical school; and graduated from college or technical school); living status (lives alone or does not live alone); and annual household income from all sources (< 15,000; 15,000- < 25,000;25,000- < 35,000; 35,000- < 50,000; and ≥ 50,000). Health care coverage was determined as any kind of health care coverage, including health insurance, prepaid plans such as health maintenance organizations (HMOs), or government plans such as Medicare, or Indian Health Service or no health care coverage.

The risk of developing ADRD and vascular dementias appears to be increased by many conditions that damage the heart and blood vessels [5]. These cognitive risk factors include heart disease, diabetes, stroke, high blood pressure and high cholesterol. If SCD respondents had ever been told by a doctor or other health care provider that they had high blood pressure, high cholesterol, coronary heart disease (angina), stroke, diabetes or heart attack (myocardial infarction), it was included as a risk factor [23, 24].

SCD subgroup data was compared with non-SCD subgroup for association with cognitive risk factors such as selected chronic conditions(summed and categorized as zero or any chronic condition) and social determinants such as education, income, living alone and access to health care within and across demographic stratum.

### Statistical analyses

To assess disparities in SCD by race and ethnicity, data were stratified by race (non-Hispanic white; non-Hispanic Black; and Hispanic). SAS 9.4 (SAS Institute Inc., Cary, NC) was used to calculate estimates with 95% confidence intervals taking into consideration the complex BRFSS sampling design. Rao -Scott chi square test was used to indicate significant differences in comparing prevalence between subgroups (*P* < .05).

## Results

### Prevalence of SCD among racial/Ethnic groups by selected characteristics (Table 1)

During 2015–2018, 10.8% (95% CI: 10.6–11.1) of adults aged 45 years or older reported SCD. The prevalence of SCD varied by race and ethnicity. SCD was reported by 10.7% (95% CI: 10.4–10.9) Whites, 12.3% (95% CI: 11.4–13.2) Blacks and 9.9% (95% CI:8.8–11.1) Hispanics (*p* = 0.0013). Across groups defined by race and ethnicity, higher percentages of adults with SCD had at least one comorbid chronic condition compared to those without SCD. More than 3 in 4 (79.1%) Blacks with SCD had at least one chronic condition compared to 60.9% without SCD (*p* < .0001). Among whites with SCD nearly 64% had at least one chronic condition as compared to less than half (46.9%) of whites without SCD (*p* < .0001). 64.1% Hispanics with SCD reported at least one chronic condition as compared to 55.5% without SCD (*p* = .0084) (Table 1).

**Table 1:** Demographic Characteristics of Adults Aged 45 Years or Older With and Without Subjective Cognitive Decline (SCD) by selected Race/Ethnic Status, Behavioral Risk Factor Surveillance System (BRFSS), 2015–2018

***N*** = 179,852						
With subjective cognitive decline *n* = 19,276				Without subjective cognitive decline *n* = 160,576		
	**Whites with SCD**			**Whites without SCD**		
	Overall	No chronic condition^a^	At least one chronic condition	Overall	No chronic condition	At least one chronic condition
	*n* = 15,172	*n* = 5346	*n* = 9826	*n* = 130,216	*n* = 65,879	*n* = 64,337
	% (95% CI)	% (95% CI)	% (95% CI)	% (95% CI)	% (95% CI)	% (95% CI)
**Overall** ^c^	10.7 (10.4–10.9)	36.1 (34.7–37.4)	63.9 (62.6–65.3)	89.3(89.1–89.6)	53.1(52.6–53.5)	46.9 (46.5–47.4)
**Sex** ^b^						
Male	47.6 (46.3–48.9)	45.6 (43.2–47.9)	48.6(46.9–50.2)	46.2(45.7–46.6)	43.3(42.7–43.9)	48.9(48.3–49.6)
Female	52.4 (51.1–53.7)	54.4 (52.1–56.8)	51.4(49.8–53.1)	53.8 (53.4–54.3)	56.7(56.1–57.3)	51.1(50.4–51.7)
**Age (y)** ^c^						
45–54	26.9 (25.7–28.3)	34.1(31.6–36.4)	23.1(21.5–24.5)	28.6 (28.1–29.1)	36.1 (35.4–36.8)	20.1(19.5–20.7)
55–64	30.1 (28.8–31.3)	30.1(27.9–32.1)	30.1 (28.6–31.7)	31.2(30.8–31.7)	32.3(31.6–32.9)	30.1(29.5–30.7)
≥ 65	42.9 (41.6–44.3)	35.9(33.8–38.2)	46.9(45.2–48.6)	40.1(39.7–40.6)	31.6(30.9–32.2)	49.8 (49.2–50.5)
**Education** ^c^						
< High School	18.7(17.3–20.1)	13.9 (12.1–15.7)	21.4(19.6–23.2)	9.2(8.9–9.6)	6.9 (6.5–7.4)	11.8 (11.3–12.3)
High School	33.1(31.8–34.4)	32.6 (30.4–34.9)	33.3 (31.7–34.9)	31.4(30.9–31.9)	29.8(29.1–30.5)	33.2(32.5–33.8)
Attended College	30.8(29.5–32.1)	31.3(29.1–33.5)	30.5(28.9–32.1)	30.6 (30.2–31.1)	30.3(29.6–30.9)	31.1(30.4–31.7)
Graduated College	17.3(16.4–18.2)	22.1(20.3–23.7)	14.6(13.7–15.6)	28.5(28.1–28.9)	32.6(32.1–33.2)	23.8(23.3–24.3)
**Income ($)** ^c^						
< 15,000	14.8(13.8–15.8)	12.4(10.8–14.1)	16.1(14.9–17.3)	5.4(5.2–5.6)	4.3(3.9–4.5)	6.7(6.4–7.1)
15,000-- < 25,000	20.8(19.5–22.1)	17.2(15.3–19.1)	22.8(21.1–24.4)	11.3(10.9–11.6)	8.7(8.4–9.1)	14.2(13.7–14.7)
25,000 - < 35,000	10.9 (10.1–11.8)	10.1(8.8–11.4)	11.4(10.2–12.5)	8.3 (8.1–8.6)	7.1(6.8–7.5)	9.7(9.3–10.1)
35,000 - < 50,000	11.2(10.4–12.1)	11.3(9.9–12.7)	11.2(10.2–12.1)	12.6(12.2–12.9)	11.8(11.3–12.3)	13.4(12.9–13.9)
≥ 50,000	27.1(25.9–28.3)	33.8(31.5–36.1)	23.3(21.9–24.7)	46.8(46.3–47.3)	52.4(51.7–53.1)	40.5(39.9–41.1)
**Living alone** ^c^						
Yes	29.2(27.7–30.7)	29.4(26.5–32.2)	29.1(27.4–30.8)	24.7(24.2–25.2)	22.4(21.7–23.2)	26.9(26.3–27.5)
**Health Care** ^c^						
No	5.9 (5.3–6.7)	6.5(5.4–7.6)	5.7 (4.8–6.5)	4.5 (4.3–4.7)	5.3 (4.9–5.6)	3.5 (3.3–3.8)
	**Blacks with SCD**			**Blacks without SCD**		
	Overall	No chronic condition	At least one chronic condition	Overall	No chronic condition	At least one chronic condition
Number	*n* = 1742	*n* = 320	*n* = 1422	*n* = 12,338	*n* = 4132	*n* = 9628
	% (95% CI)	% (95% CI)	% (95% CI)	% (95% CI)	% (95% CI)	% (95% CI)
**Overall** ^c^	12.3(11.4–13.2)	20.9(17.7–24.2)	79.1(75.8–82.3)	87.7(86.8–88.6)	39.1(37.7–40.5)	60.9(59.5–62.3)
**Sex**						
Male ^b^	42.3(38.4–46.3)	37.4(29.2–45.6)	43.5 (39.1–47.9)	45.9(44.4–47.5)	47.1(44.6–49.7)	45.2(43.3–47.2)
Female	57.7(53.7–61.6)	62.6(54.4–70.8)	56.5 (52.1–60.9)	54.1(52.5–55.6)	52.9(50.3–55.4)	54.8(52.8–56.7)
**Age (y)**						
45–54	37.5(33.5–41.6)^c^	49.3(40.7–57.9)^b^	34.4(30.1–38.8)^c^	34.3(32.8–35.8)	45.8 (43.5–48.2)	26.9(25.1–28.8)
55–64	32.1 (28.4–35.6)^c^	26.9(19.5–34.4)^b^	33.4(29.4–37.4)^c^	34.2(32.8–35.6)	32.4(30.2–34.5)	35.3(33.6–37.1)
≥ 65	30.4(27.1–33.8)^c^	23.7(16.2–31.2)^b^	32.2 (28.5–35.9)^c^	31.5(30.2–32.8)	21.8(20.1–23.5	37.7 (36.1–39.4)
**Education** ^c^						
< High School	30.8(26.8–34.7)	24.7(15.6–33.8)	32.4(28.1–36.6)	19.5(18.2–20.7)	13.7 (11.9–15.6)	23.1(21.5–24.8)
High School	29.5(26.1–32.9)	32.9(25.2–40.5)	28.6(25.1–32.3)	32.2(30.8–33.6)	31.7(29.5–33.9)	32.5(30.8–34.2)
Attended College	27.8(24.2–31.4)	26.4(18.9–33.9)	28.2(24.2–32.2)	28.6 (27.2–30.1)	29.4(27.1–31.6)	28.1(26.4–29.9)
Graduated College	11.9(9.8–14.1)	15.9(11.1–20.8)	10.8(8.5–13.1)	19.7(18.6–20.8)	25.2(23.2–27.1)	16.2(14.9–17.3)
**Income ($)** ^c^						
< 15,000	31.5(27.4–35.5)	26.2(18.1–34.3)	32.9(28.3–37.4)	17.7(16.6–18.9)	13.1(11.4–14.7)	20.8(19.2–22.4)
15-- < 25,000	30.4(26.5–34.3)	26.9(19.1–34.8)	31.3(27.1–35.5)	24.5(23.1–25.9)	22.1(19.8–24.2)	26.1(24.3–27.9)
25,000 - < 35,000	11.1(8.5–13.7)	7.7(3.7–11.6)	12.1(8.9–15.1)	11.8(10.8–12.7)	10.9 (9.4–12.4)	12.3(11.1–13.6)
35,000 - < 50,000	9.5(6.5–12.4)	11.6(4.4–18.8)	8.9(5.7–12.1)	12.8(11.7–13.9)	12.9(11.2–14.7)	12.7(11.3–14.1)
≥ 50,000	17.5(13.9–21.1)	27.6(18.3 36.9)	14.9(11.3–18.5)	33.2(31.6–34.7)	41.1(38.4–43.7)	28.1(26.2–29.8)
**Living alone**						
Yes	37.8(32.9–42.7^c^	33.5(22.5–44.5)^b^	38.8(33.8–43.8^c^)	31.3(29.8–32.8)	30.4(27.9–32.9)	31.7(29.9–33.5)
**Health Care**						
No	11.5(8.8–14.3)^b^	17.5(10.5–24.6)^c^	9.9(7.5–12.4)^b^	9.1(8.2–10.1)	10.9(9.3–12.6)	7.9(6.9–8.9)
	**Hispanics with SCD**			**Hispanics without SCD**		
	Overall	No chronic condition	At least one chronic condition	Overall	No chronic condition	At least one chronic condition
Number	*n* = 794	*n* = 261	*n* = 533	*n* = 7215	*n* = 2968	*n* = 4247
	% (95% CI)	% (95% CI)	% (95% CI)	% (95% CI)	% (95% CI)	% (95% CI)
**Overall**	9.9(8.8–11.1)	35.9(30.2–41.8)	64.1(58.2–69.8)	90.1 (88.9–91.2)	44.5(42.6–46.3)	55.5(53.6–57.4)
**Sex** ^b^						
Male	48.5(42.7–54.3)	48.7(38.9–58.6)	48.4(41.3–55.5)	45.9(44.1–47.8)	49.1(46.1–52.1)	43.8(41.6–46.1)
Female	51.4(45.7–57.3)	51.3 (41.4–61.1)	51.6(44.5–58.7)	54.1(52.2–55.9)	50.9(47.9–53.9)	56.2(53.9–58.4)
**Age (y)** ^b^						
45–54	40.8(34.8–46.8)	55.6(45.8–65.4)	32.4(26.1–38.8)	42.6(40.6–44.5)	55.5(52.4–58.6)	32.2 (30.1–34.3)
55–64	33.1(27.7–38.5)	25.3(18.3–32.4)	37.5(30.7–44.3)	28.7(27.1–30.5)	25.9(23.3–28.5)	31.1(28.8–33.2)
≥ 65	26.1(21.2–31.1)	19.1(12.9–25.3)	30.1(24.1–36.1)	28.7(27.1–30.3)	18.6(16.1–21.1)	36.8(34.8–38.8)
**Education**						
< High School	46.7(40.6–52.9)^c^	47.2(36.4–57.9)^b^	46.4(39.3–53.5)^c^	39.3(37.3–41.3)	37.8(34.3–41.4)	40.5(38.2–42.8)
High School	22.7(18.2–27.1)^c^	25.4(17.1–33.7)^b^	21.1(16.1–26.2)^c^	24.3 (22.8–25.9)	24.4(21.8–26.9)	24.3(22.5–26.1)
Attended College	20.4(16.3–24.6)^c^	16.3(10.8–21.8)^b^	22.7(18.2–27.3)^c^	20.5(19.1–21.9)	21.5 (18.9–24.1)	19.7(18.1–21.4)
Graduated College	10.2(7.8–12.6)	11.2(7.1–15.3)	9.7(6.7–12.6)	15.9(14.8–16.9)	16.3(14.6–17.9)	15.5(14.4–16.7)
**Income**						
< 15,000	37.1(30.6–43.5)^c^	36.4(24.9–47.7)^c^	37.4(29.9–44.9)^b^	30.6(28.8–32.4)	22.2(19.4–24.9)	37.4(35.1–39.7)
15-- < 25,000	29.5(23.6–35.4)^c^	^d^	31.8(24.8–38.9)^b^	25.2(23.4–26.9)	21.9(19.2–24.5)	27.9(25.7–30.2)
25,000 - < 35,000	11.9(7.9–15.9)^c^	12.9(7.8–18.1)^c^	11.2(5.6–16.8)^b^	12.4(10.7–14.1)	14.7(11.6–17.8)	10.5(8.9–11.9)
35,000 - < 50,000	8.1(4.5–11.7)^c^	6.8(3.2–10.4)^c^	8.9 (6.5–11.4)^b^	11.3(9.9–12.7)	14.4(11.9–16.7)	8.8(7.3–10.4)
≥ 50,000	13.4(9.2–17.7)^c^	18.1(9.6–26.5)^c^	10.6(6.4–14.8)^b^	20.5(18.9–22.1)	26.8(24.1–29.7)	15.4(13.8–16.9)
**Living alone** ^b^						
Yes	29.7(23.2–36.1)	^d^	25.9(19.7–32.2)	23.5(21.3–25.6)	22.8(18.8–26.8)	23.8(21.7–26.1)
**Health Care** ^b^						
No	14.8(9.5–20.2)	^d^	12.9(6.6–19.3)	14.1(12.7–15.4)	18.5(16.2–20.8)	10.5(8.9–12.1)

*Note*: frequencies presented are unweighted. Percentages and confidence intervals are weighted based on state population sizes

^a^ selected chronic conditions or cognitive risk factors - angina, heart attack, stroke, high cholesterol, high blood pressure and diabetes

^b^ *not significant* compared to group without SCD

^c^ *significant* compared to group without SCD

^d^ Data suppressed (confidence interval more than 20 points wide)

There was a higher percentage of females in both subgroups- SCD and without SCD across race/ethnicity. However, differences by gender were not significant. Whites with SCD showed a significantly higher proportion of 65 and older age group as compared to whites without SCD (*p* = .0009). Interestingly, more whites without SCD in the 65 and older age group had at least one comorbid chronic condition (49.8%) as compared to whites with SCD (46.9%) (*p* = .001). In Blacks with and without SCD, 45–54-year age group had the highest percentage, 37.5 and 34.3% respectively, although the difference was not significant(0.27). However, in the 45–54-year age group, Blacks with at least one comorbid chronic condition (34.4%) were significantly higher in the SCD subgroup compared to without SCD (26.9%) (*p* = .0017). Hispanics with and without SCD had the highest prevalence in the 45–54 years age group, 40.8 and 42.6% respectively,, though not significant (*p* = 0.288). Differences in prevalence of at least one chronic condition by age group did not show significant variation in Hispanics with and without SCD (Table 1).

Whites with SCD, overall and with at least once chronic condition were less likely to be educated than without SCD (*p* < .0001). Similar differences were observed among both Blacks and Hispanics with lower educational attainment in groups with SCD than without. In addition, Hispanics and Blacks with SCD were more likely to be less educated compared to whites across all selected educational categories (*p* < .0001). Amongst Hispanics with SCD, 46.7% (95% CI: 40.6–52.9) and amongst Blacks with SCD, 30.8% (95% CI: 26.8–34.7) were less than high school educated compared to 18.7% (95% CI: 17.3–20.1). More whites with SCD were found to be college graduates 17.3% (95% CI: 16.4–18.2) as compared to Blacks 11.9% (95% CI: 9.8–14.1) and Hispanics 10.2% (95% CI: 7.8–12.6) (Table 1).

Significant income differences were also noted. Amongst all racial/ethnic groups with SCD overall, and with at least one chronic condition, income was significantly lower as compared to group without SCD. 40.5% whites without SCD had an income of ≥ $50,000 as compared to 27.1% Whites with SCD (*p* < .0001). 23.3% whites with SCD and at least one chronic condition reported an income of ≥ $50,000 compared to 52.4% Whites without SCD and with at least one chronic condition(*p* < .0001). Only 17.5 Blacks with SCD overall, showed an income of ≥ $50,000 compared to 33.2% without SCD(*P* < .0001). In the presence of at least one chronic condition too, more Blacks (28.1%) without SCD earned ≥ $50,000 compared to Blacks with SCD (14.9%) (*P* < .0001). In comparison across racial groups, disparities were evident. Whites with SCD were more likely to have a higher income. More whites reported an income of $50,000 or above, 27.1% (95% CI: 25.9–28.3) compared to Blacks 17.5% (95% CI: 29.6–44.9) and Hispanics 13.4% (95% CI: 9.2–17.7). Both Hispanics,37.1% (95% CI: 30.6–43.5) and Blacks 31.5% (95% CI: 27.4–35.5) with SCD earned < $15,000 compared to whites with SCD,14.8% (95% CI: 13.8–15.8) (*p* < .0001) (Table 1).

Amongst all racial/ ethnic groups, number of adults living alone with SCD was higher than the number of adults living alone without SCD. 29.2% whites with SCD lived alone as compared to 24.7% of whites without SCD (*p* < .0001). In whites with SCD and reporting at least one chronic condition 29.1% reported living alone compared to 26.9% without SCD and with at least one chronic condition(*p* = .016). Amongst Blacks with SCD, 37.8% lived alone compared to 31.3% Blacks without SCD (*p* = .0108). 38.8% Blacks with SCD and reporting at least one chronic condition mentioned living alone as compared to 31.7 Blacks without SCD and reporting at least one chronic condition (*p* = .0073). In Hispanics with SCD, 29.6% lived alone compared to 23.5% Hispanics without SCD (*p* = .0548). Similarly, more Hispanics with SCD and with at least one chronic condition (25.9%) lived alone compared to Hispanics without SCD and with at least one chronic condition (23.8%) (*p* = 0.5238). Overall, Blacks with SCD were more likely to be living alone. Amongst Blacks with SCD, 37.8% lived alone compared to Hispanics 29.6%% and Whites, 29.2% (*p* < 0.0008) (Table 1).

Health care coverage differences were also observed by race/ethnicity in groups with and without SCD. More whites with SCD (5.9%) reported no health care coverage as compared to 4.5% Whites without SCD (*p* < .0001). Similarly, more whites with SCD and at least one chronic condition (5.7%) had no health insurance compared to whites without SCD and at least one chronic condition (3.5%) (*p* < .0001). Blacks with SCD and Blacks with SCD plus at least one chronic condition too, had a higher percentage of uninsured compared to corresponding non SCD group but these differences were not significant. Similar results were reported for Hispanics, with more uninsured in the SCD group as compared to non SCD group but not being significant. Overall, more Hispanics (14.8 95% CI: 9.5–20.1) and Blacks (11.5 95% CI: 8.8–14.3) with SCD were likely to have no health insurance compared to whites with SCD (5.9 95% CI: 5.3–6.7) (*p* < .0001) (Table 1).

### Prevalence of selected chronic conditions (cognitive risk factors) among racial/Ethnic groups with SCD (Table 2)

Prevalence of chronic conditions (cognitive risk factors) was significantly higher in all racial and ethnic groups with SCD as compared to without SCD. This was true for all selected cognitive risk factors (angina, heart attack, stroke, high cholesterol, high blood pressure and diabetes) examined in this study. Top three chronic conditions with the highest prevalence for adults with SCD were high cholesterol, high blood pressure and diabetes amongst all racial/ethnic groups (Table 2).

**Table 2:** Disparities in Selected Chronic Conditions (cognitive risk factors) among selected Racial/Ethnic groups with and without Subjective Cognitive Decline (SCD)

	White, Non-Hispanic	Black, Non-Hispanic	Hispanic
**Cognitive Risk Factor**	**% (95% Confidence Interval)**	**% (95% Confidence Interval)**	**% (95% Confidence Interval)**
**Angina**			
With SCD	16.2(14.8–17.4)	13.2(9.6–16.8)	19.3(12.6–25.9)
Without SCD	7.1(6.8–7.4)	6.5(5.5–7.5)	6.7 (5.6–7.8)
Prevalence Ratio	2.3(2.1–2.5) ^a^	2(1.5–2.8) ^a^	2.9(2–4.2) ^a^
**Heart Attack**			
With SCD	15.4(14.1–16.8)	13.7(10.3–17.1)	12.8(7.4–18.2)
Without SCD	6.6 (6.3–6.9)	6.7(5.7–7.7)	6.2(5.2–7.2)
Prevalence Ratio	2.3 (2.1–2.6)^a^	2(1.5–2.7) ^a^	2.1(1.3–3.3) ^b^
**Stroke**			
With SCD	13.6(12.3–14.9)	14.3(11.1–17.6)	10.4(5.4–15.3)
Without SCD	4.1(3.8–4.3)	7.2 (6.2–8.2)	3.4(2.6–4.2)
Prevalence Ratio	3.4 (3–3.8) ^a^	2(1.5–2.6) ^a^	3.1(1.8–5.1)
**High Cholesterol**			
With SCD	60.7(58.9–62.6)	65.3(60.5–70.1)	60.4(52.6–68.3)
Without SCD	46.1(45.4–46.7)	45.2(43.3–47.1)	44.6(42.3–46.9)
Prevalence Ratio	1.3(1.3–1.4)^a^	1.4(1.3–1.6) ^a^	1.4(1.2–1.6) ^c^
**High Blood pressure**			
With SCD	60.3(58.4–62.1)	78.9(74.6–83.2)	60.1(51.9–68.2)
Without SCD	48.2(47.5–48.8)	66.3(64.4–68.1)	52.5(50.3–54.7)
Prevalence Ratio	1.3(1.2–1.3)^a^	1.2(1.1–1.3) ^a^	1.1(1–1.3) ^d^
**Diabetes**			
With SCD	26.7(25.1–28.4)	37.5(32.7–42.4)	40.5 (32.2–48.7)
Without SCD	15.4(14.9–15.9)	27.2(25.5–28.9)	25.8 (23.8–27.8)
Prevalence Ratio	1.7 (1.6–1.9)^a^	1.4(1.2–1.6) ^a^	1.6(1.3–1.9) ^e^

^a^ *p* < .0001 ^b^ *p* = 0.0018 ^c^ *p* = 0.0002 ^d^ *p* = 0.0846 ^e^ *p* = 0.0002

Amongst whites with SCD, there was a significantly higher prevalence of chronic conditions compared to those without SCD (*p* < .0001). The prevalence of several conditions was at least twice among Whites with SCD compared to those without, including angina (PR = 2.3, 95% CI: 2.1–2.5), heart attack (PR = 2.3, 95% CI: 2.1–2.6), and stroke (PR = 3.4, 95% CI: 3–3.8) (Table 2).

Similarly, prevalence of chronic conditions among Blacks with SCD was significantly higher than among those without SCD (*p* < .0001 for all conditions). The prevalence of angina (PR = 2, 95% CI: 1.5–2.8), heart attack (PR = 2, 95% CI: 1.5–2.7), and stroke (PR = 2 95% CI: 1.5–2.6), were two times higher among Blacks with SCD compared to the non SCD group (Table 2).

In Hispanics too, the prevalence of chronic condition risk factors was significantly higher for stroke (PR = 3.1, 95% CI: 1.8–5.1), angina (PR = 2.9, 95% CI: 2–4.2), heart attack (PR = 2.1, 95% CI: 1.3–3.3), high cholesterol (PR = 1.4, 95% CI: 1.2–1.6), and diabetes (PR = 1.6, 95% CI: 1.3–1.9) among those with SCD compared to those without SCD (Table 2).

### Prevalence of functional limitations among racial/Ethnic groups with SCD (Table 3)

By race and ethnicity, Hispanics and Blacks with SCD were more likely to report SCD-related functional limitations compared to whites (*p* < .001) (Table 3).

**Table 3:** Disparities in Functional Limitations ^a^ among Racial/Ethnic groups with Subjective Cognitive Decline (SCD)

	White, Non-Hispanic	Black, Non-Hispanic	Hispanic
	% (95% Confidence Interval)	% (95% Confidence Interval	% (95% Confidence Interval
**Given up day-to-day chores due to confusion or memory loss ^**			
Yes	37 (35.6–38.4)	53.7(49.7–57.7)	56.6(50.8–62.4)
**Need assistance with day-to_day activities due to confusion or memory loss ^**			
Yes	31.4 (30.1–32.7)	47.4 (43.4–51.3)	51.1(45.1–57.1)
Need help with day-to-day activities are you able to get it *			
Yes	87.2(85.8–88.7)	85.9(81.9–90.1)	87.2(82.5–91.9)
**Confusion or memory loss interfere with work or social activities^**			
Yes	34.2(32.9–35.6)	48.2(44.2–52.3)	40.9(35.3–46.5)
Discussed your confusion or memory loss with a health care professional *			
No	53.2(51.7–54.5)	53.2(49.1–57.2)	55.5(49.7–61.4)
**SCD related functional limitations** ^**a**^ **^**			
Yes	45.9 (44.5–47.2)	63.5(59.7–67.3)	63.5(57.9–69.1)

^a^ Defined as the presence of either of a respondent reporting that SCD always, usually, or sometimes (1) caused them to give up household chores or activities or (2) interfered with their ability to work, volunteer, or engage in social activities outside the home

**^** *p* < .0001 * not significant

Nearly 57% (95% CI: 50.8–62.4) Hispanics and 54% (95% CI: 49.7–57.7) Blacks with SCD reported having to always, usually, or sometimes give up household activities because of SCD compared to 37% (95% CI: 35.6–38.4) whites (*p* < .0001). Only 31.3% (95% CI: 30.1–32.7) of whites compared to 47.4% (95% CI: 43.4–51.3) of Blacks and 51.1% (95% CI: 45.1–57.1) of Hispanics always, usually, or sometimes needed assistance with day-to_day activities due to confusion or memory loss (*p* < .0001) (Table 3).

Confusion or memory loss always, usually, or sometimes interfered with work or social activities in all racial and ethnic groups. Interference with work or social activities was highest in Blacks with SCD (48.2 95% CI: 44.2–52.3) followed by Hispanics (40.9%95% CI: 35.3–46.5) and Whites (34.2 95% CI: 32.9–35.6) (*p* < .0001). Overall, of those with SCD, 63.5% (95%CI: 59.7–67.3) Blacks and 63.5% (95%CI: 57.9–69.1) Hispanics reported functional limitations as compared to Whites 45.9% (95% CI: 44.5–47.2) (Table 3).

The percentage of those who discussed SCD with a health care professional also varied by race and ethnicity although the differences were not significant ((*p* = 0.7319). More than half of respondents across race/ethnicity did not report discussing SCD with a health care professional (Table 3).

## Discussion

Regardless of race/ethnicity, SCD prevalence was associated with differences in comorbid chronic conditions and social determinants. Adults with SCD not only had two or three times the prevalence of some chronic conditions but were also disproportionately affected by social determinants compared to those without SCD. Study findings demonstrated several adverse determinants such as poverty, low education, access to health care and isolation compounded by a higher prevalence of chronic conditions across racial/ethnic subgroups with SCD compared to without SCD.

Previous work has identified statistically significant associations between each of the above listed social determinants and risk for ADRD [25–28]. Prior research has also elucidated the complications in management of comorbid chronic conditions [17] and association with a higher risk of developing Alzheimer’s or other dementias [5, 16, 29] in persons with SCD. Cognitive dysfunction in areas of learning, memory, and decision-making increase the need for assistance in chronic disease self-management, such as help with medication schedule or medical appointments. Presence of comorbid chronic condition or its treatment may also result in a missed or delayed SCD consult as memory problems could be attributed to the chronic condition or treatment side-effects If a person has memory problems co-occurring with a chronic condition or treatment, cognitive dysfunction might be regarded as related to the condition or the treatment and not to early stages of ADRD. The patient may not report these symptoms or if reported, the provider may misattribute them to medication side effect [16, 17, 29].

SCD is a concern, by itself. It is also poignant that measures, which are necessary to manage SCD and reduce the risk for progression to ADRD, are maybe exponentially more difficult for those with both adverse social determinants and comorbid chronic conditions. Within this context, disparities by race were evident. Blacks and Hispanics with SCD were disproportionately affected by social inequalities and comorbid chronic conditions compared to whites with SCD. These two minority groups with SCD were more likely to be less educated, had lower annual household incomes and lack of access to health care as compared to whites with SCD. In contrast to the white subgroup with SCD, Hispanics and Blacks with SCD also reported a greater likelihood of living alone. Individuals with cognitive impairment who live alone are more vulnerable [27, 28]. They may have an increased risk for injury to self or others and report more unmet needs such as managing money, medications, mobility, and some activities of daily living. Older adults who live alone are less likely to use health services and are at a risk for poor health outcomes [27, 28]. In addition, Hispanics and Blacks also reported highest prevalence of SCD in the relatively younger, 45–54-year age group as compared to Whites. Irrespective of the reasons, it is safe to comment on the health and financial consequences ensuing from a higher SCD prevalence in the less than 55 years age group. Adults aged 45–54 years are at the peak of their careers regards salary, productivity and contributions to their retirements [17, 18]. SCD may lead to functional limitations that have substantial social and economic impact on these individuals and their families [18, 30].

High cholesterol, high blood pressure and diabetes were the most prevalent chronic conditions across racial/ethnic groups in this study. Blacks with SCD had the highest prevalence of high blood pressure while Hispanics with SCD led the prevalence for diabetes. A significantly higher burden of chronic conditions was evident in Blacks with SCD- close to 80 % reported at the least one chronic condition.

Significant disparities in functional limitations were noted by race and ethnicity among those with SCD. Functional impairment in activities of daily living has been documented in prior studies of older adults with cognitive impairment [17, 18, 31, 32]. Population-based estimates have reported weighted prevalence of ≥1 functional limitation varying from 45% [31] and 51% [18] to 57% [33]. Hispanics and Blacks had a higher prevalence of SCD-related limitations that affected work, household chores, or social activities as compared to whites. Functional limitations predict significant increase in support from formal and/or informal caregiver resources [33, 34]. This care represents a significant time commitment for families and a significant economic cost to society [33].

Less than half of Blacks (46.8%) and Hispanics (44.5%) with SCD had discussed their cognitive decline with a health-care provider. Given the association of AD with dependence and disability for a long duration, the earlier the detection, the sooner people and their families can receive information regarding better management. The benefits of early assessment, diagnosis, and disclosure of that diagnosis are well known, so efforts focused on care navigation and culturally competent physician-initiated conversations on cognitive health are recommended [35]. Opportunities for improvement could also build on the frequent interactions amongst vulnerable age groups and nonphysician care providers. Trusted culture -sensitive relationship with health care providers could encourage older adults and their families to express concerns about the older adult’s cognition facilitating earlier diagnosis of dementia [36]. It is interesting to note that Healthy People 2030, a vital framework for prioritizing health issues in U.S. has included a new objective of increasing the proportion of adults with SCD who discuss their confusion or memory loss with a health-care professional by five percentage points over the next decade [37].

This study is subject to several limitations. First. BRFSS is a telephone survey that samples from noninstitutionalized adults. People living in nursing homes and long-term care facilities without cell phones assigned by a phone company may not be included. It is likely that both SCD and chronic conditions are more common among older adults living in these institutions. Second, SCD by definition is self-reported, and not an objective assessment. Third, it may underrepresent people who have severe cognitive impairment because of the functional capacity required to participate in the survey. Additionally, BRFSS measure of SCD asks only about changes in memory or thinking which may not capture the full domain of cognitive dysfunction [22]. Despite these limitations, the BRFSS is a uniquely powerful tool to provide the national prevalence of cognitive decline and related health issues among racial and ethnic groups, due to its large sample size and proven reliability and validity.

## Conclusion

In lieu of an aging U.S. population, SCD is a critical public health issue. Of particular concern is the demographic projection for aging minority populations and anticipated rise in burden of ADRD. Given the gravity of this situation, it is vital to put a spotlight on cognitive impairment related disparities that may exist among minority communities. Population-based measures of SCD can be extremely useful to the public health community, health care providers, and policymakers in prioritization and planning efforts [38]. This study uses population data to explore the profile of ethnoracial minority groups with SCD. Notable findings include a significantly higher burden of chronic conditions and adverse social determinants among Blacks and Hispanics with SCD, predicting worse cognitive health outcomes. Not surprisingly, SCD associated functional limitations were also found to be significantly higher in Hispanics and Blacks compared to whites, foretelling a need for increased support from caregivers. Moreover, less than 50% of the minority groups had discussed their SCD with a provider. Healthy People 2030’s new objective to increase the proportion of adults with SCD who discuss their confusion or memory loss with a health-care professional by five points encourages ongoing analyses of SCD data. It would be interesting to measure progress towards this goal amongst minority groups over time. Study results are expected to help public health stakeholders make data -driven decisions and be better prepared for the challenges associated with disparities in cognitive decline. Lastly, this study also serves to inform and bolster clinical SCD related research in minority subgroups. Population -level results as presented in this study can guide future researchers in targeted study design and sampling from vulnerable groups.

## Data Availability

Publicly available data from the Centers for Disease Control and Prevention. The datasets generated and/or analyzed during the current study are available as follows: Data file 2015: https://www.cdc.gov/brfss/annual_data/annual_2015.html Data file 2016: https://www.cdc.gov/brfss/annual_data/annual_2016.html Data file 2017: https://www.cdc.gov/brfss/annual_data/annual_2017.html Data file 2018: https://www.cdc.gov/brfss/annual_data/annual_2018.html

## Abbreviations

SCD: Subjective Cognitive Decline
AD: Alzheimer’s Disease
BRFSS: Behavioral Risk Factor Surveillance System
ADRD: Alzheimer’s Disease and Related Dementia
SDOH: Social Determinants of Health
CDC: Centers for Disease Control and Prevention
DC: District of Columbia
